# How I Do It: A Stepwise Surgical Technique for Fisch Class B Glomus Tympanicum

**DOI:** 10.1002/lary.70467

**Published:** 2026-03-05

**Authors:** Stéphane Gargula, Dario Ebode, Denis Ayache, Thomas Radulesco, Mary Daval, Giannicola Ianella, Maria‐Pia Tuset, Ralph Haddad, Justin Michel

**Affiliations:** ^1^ ENT‐HNS Department Aix Marseille Univ, APHM, CNRS, IUSTI, La Conception University Hospital Marseille France; ^2^ ENT‐HNS Department, Hospital Fondation Adolphe de Rothschild Paris France; ^3^ Department of Organi Di Senso University Sapienza Rome Italy

**Keywords:** ear neoplasms/surgery, glomus tympanicum tumor, middle ear/surgery, paraganglioma/surgery

## Abstract

We describe a stepwise retroauricular microsurgical workflow for bulky tympanic paragangliomas, combining circumferential canaloplasty with temporary removal and replacement of the tympanomeatal flap and malleus to optimize exposure and bleeding control. This strategy facilitates early identification of critical structures, stepwise devascularization, and safe tumor excision in bulky lesions extending to the hypotympanum or protympanum. The technique emphasizes operative sequencing and surgical ergonomics in challenging cases.

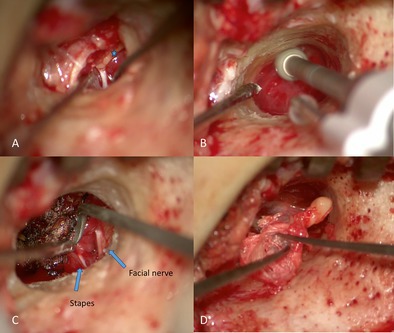

## 1 Introduction

Tympanic paragangliomas (glomus tympanicum) are benign but highly vascular middle ear tumors [1, 2]. While endoscopic or microscope‐assisted transcanal surgery is effective for class A and some selected class B1 tumors, larger Fisch class B lesions often extend to the hypotympanum and protympanum, where exposure and bleeding control are more challenging [3, 4, 5]. Although Fisch class B tumors are defined by mastoid extension, surgical strategy is often guided by tumor volume and middle ear involvement, and bulky class A lesions may require a similar workflow.

We describe a retroauricular microsurgical workflow combining circumferential canaloplasty with temporary removal and replacement of the tympanomeatal flap and malleus, intended to optimize exposure and facilitate controlled tumor excision in selected Fisch class B tympanic paragangliomas. A didactic Video 1 is provided to illustrate each operative step and highlight technical nuances. The purpose of this report is not to introduce a novel surgical approach, but to formalize and illustrate a structured operative strategy for these lesions.

**VIDEO 1:** Step‐by‐step microsurgical excision of a bulky tympanic Paraganglioma using a retroauricular approach with circumferential canaloplasty and temporary tympanomeatal flap and malleus mobilization. Video content can be viewed at https://onlinelibrary.wiley.com/doi/10.1002/lary.70467.

## 2 Methods

Written informed consent was obtained from the patient for the recording, use, and online dissemination of surgical images and video material. The procedure is performed under general anesthesia with orotracheal intubation. The patient is placed supine with the head turned to the contralateral side, and facial nerve monitoring is systematically used. A retroauricular incision is performed, followed by elevation of an anteriorly based musculoperiosteal flap. A circular incision of the external auditory canal is made, and a circumferential canaloplasty is carried out using a 3‐mm coarse diamond burr with particular attention to the inferior quadrant, in order to widen the bony meatus and optimize visualization of the hypotympanum. The extent of drilling is adapted to individual anatomical variations, including the prominence of the anterior canal wall and the relative depth of the hypotympanum, with the objective of exposing the entire circumferential surface of the tumor as it molds the tympanic cavity.

**FIGURE 1:**
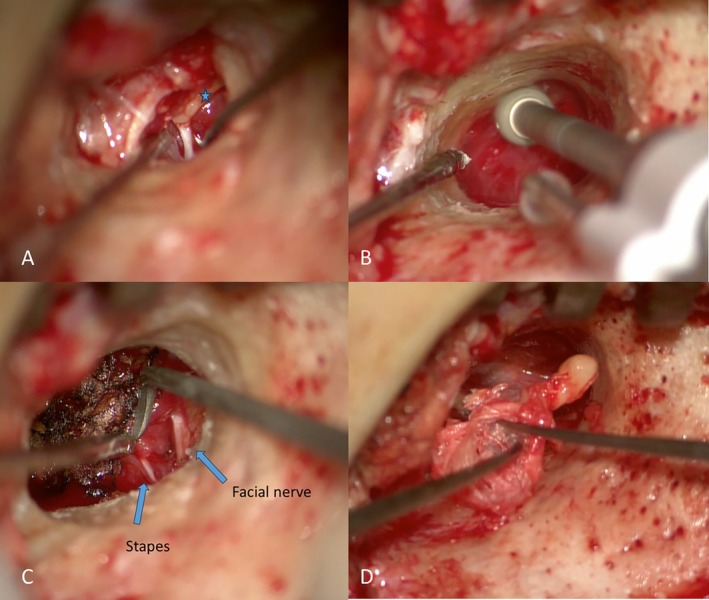
Key steps of the technique. (A) The long process of the incus (starred) is followed for early incudostapedial disarticulation. (B) After removal of the tympanomeatal flap, canaloplasty must allow full access to the tumor before any manipulation. (C) The tumor surface is cauterized with a diode laser and mobilization begins at the superior pole. (D) The tympanomeatal flap is repositioned after tumor excision.

A Rosen notch is then created to allow incudostapedial disarticulation, thereby securing the inner ear early in the procedure (Figure 1). The chorda tympani can occasionally be preserved, but this is a secondary goal in large lesions. The long process of the incus is usually easy to identify and can be followed down to the stapes, which is frequently hidden underneath the tumor. The incus is then removed and stored for potential ossiculoplasty.

For bulky tumors, we routinely perform a complete remove‐and‐replace maneuver of the tympanomeatal flap together with the malleus, which represents a central component of the operative strategy described here. During removal of the tympanomeatal flap with the malleus, the tensor tympani tendon may be sectioned under direct vision or, when embedded in the tumor, released using a blunt instrument or during mobilization of the malleus. The entire flap, including the tympanic membrane and malleus handle, is detached “en bloc” and placed on a speculum, kept moist in sterile gauze. Beyond exposure, the primary rationale for this maneuver is to protect the tympanic membrane and to allow rapid tumor mobilization and extraction in hemorrhagic situations. This provides an unobstructed view of the mesotympanum and hypotympanum, allowing controlled tumor dissection with minimal risk to the tympanic membrane. An additional advantage of this configuration is that suction can be performed with wider‐caliber aspirators if bleeding is profuse, without the risk of damaging the tympanomeatal flap. Depending on the extent and superior spread of the lesion, additional exposure may be required. In cases involving the attic or mastoid antrum, a transmeatal atticotomy completed by a limited mastoidectomy can be performed to fully expose the tumor before starting the excision.

Excision begins with surface coagulation of the tumor using a fiber‐delivered diode laser in pulsed mode. In contrast to stapes surgery, the interval between pulses can be reduced to accelerate tissue coagulation when working at a safe distance from the facial nerve and the stapes. Laser use is deliberately limited in close proximity to these structures. This stiffens and shrinks the mass, facilitating mobilization and reducing bleeding induced by tears. Dissection is initiated at the superior pole, which usually contains fewer vascular pedicles but lies close to the most critical structures: the tympanic segment of the facial nerve and the stapes. In this area, laser use is minimized to avoid iatrogenic injury. As the dissection proceeds toward the hypotympanum, the laser is used more extensively to coagulate feeding pedicles and adhesions, while the tumor is gently mobilized forward and upward.

Significant bleeding may arise from intraosseous pedicles, sometimes arterial in origin. In these cases, bipolar cautery and laser are often ineffective. Bone wax, or a diamond burr used without irrigation, can be more effective for hemostasis. Because all critical structures have already been secured before addressing this most hemorrhagic quadrant, the tumor can be removed rapidly if bleeding becomes difficult to control. Once the mass is out, visualization improves and hemostasis becomes much easier. Excision is usually completed by removing the tumor portion extending into the Eustachian tube, which is generally straightforward at this stage as the lesion is already devascularized.

Reconstruction begins with placement of a hammock of temporalis fascia, leaving a notch superiorly to accommodate the malleus neck. The tympanomeatal flap is carefully lifted from the speculum, rehydrated, and re‐draped in its anatomical orientation. The annulus is meticulously repositioned into the sulcus, particularly anteroinferiorly to prevent blunting. When extensive canaloplasty has enlarged the bony diameter, counter‐incisions may be required to adapt the flap onto the fascia.

Ossicular reconstruction is most often performed by incus transposition, which does not require cartilage reinforcement of the tympanic membrane, although alternative techniques may be considered. Stabilization of the flap is obtained with two thin silicone sheets, ensuring the essential preservation of a sharp anterior angle, and completed with a beveled oto‐wick. Because a circumferential incision of the canal skin has been made, meticulous repositioning is crucial to avoid bone exposure and impaired healing. The silastic sheets are first inserted inside the oto‐wick and, after closure of the posterior incision, gently pulled back into the external auditory canal. This prevents interposition between the superficial canal skin and the bony canal wall. A second oto‐wick is then placed to secure the superficial external auditory canal skin. The described workflow reflects a shared practice between the participating centers.

## 3 Key Technical Points

This workflow emphasizes early identification of key structures, including the stapes and facial nerve, and a stepwise devascularization strategy to facilitate controlled dissection. The wide exposure obtained after tympanomeatal flap and malleus mobilization supports management of bleeding from hypotympanic pedicles and allows rapid removal of the mass when needed.

## 4 Discussion

Endoscopic and transcanal microscopic approaches are well established for class A and selected small class B tympanic paragangliomas, offering excellent visualization with limited morbidity. These techniques are particularly effective when tumor volume is limited and bleeding can be readily controlled within the confines of the external auditory canal [3, 4]. However, in larger lesions that mold the tympanic cavity or extend anteriorly or inferiorly toward the hypotympanum or protympanum, exposure and bleeding control may become more challenging. Limited working space, reduced suction efficiency, and traction on the tympanic membrane can complicate tumor mobilization in these situations. In this context, a retroauricular workflow combining circumferential canaloplasty with temporary mobilization of the tympanomeatal flap and malleus prioritizes wide exposure and early protection of critical middle ear structures. This configuration allows unobstructed access to the entire tumor surface, facilitates stepwise devascularization, and enables rapid tumor extraction when required, particularly in hemorrhagic settings.

Overall, this strategy emphasizes operative sequencing and surgical ergonomics rather than reliance on a specific instrument or technology. This report focuses on operative exposure and technical strategy in challenging cases, rather than on outcome‐based measures. The accompanying video illustrates the key steps and technical nuances of this workflow and is intended to support surgical decision‐making in selected cases.

## Funding

The authors have nothing to report.

## Ethics Statement

This technical report illustrates a standard surgical procedure performed in accordance with institutional and national ethical standards. According to French regulations, Institutional Review Board (IRB) approval is not required for descriptive surgical technique papers that do not involve research on human subjects. The patient provided written informed consent for the use of surgical images and video material for publication.

## Conflicts of Interest

The authors declare no conflicts of interest.

## Supporting information


**Data S1:** lary70467‐sup‐0001‐Supinfo.pdf.

## Data Availability

All relevant data are within the paper.
