# Supplementary material for: How I Do It: A Stepwise Surgical Technique for Fisch Class B Glomus Tympanicum
Source: Laryngoscope. 2026 Mar 5;136(7):3225–8. doi: 10.1002/lary.70467 (PMC13253175; doi:10.1002/lary.70467)
Supplement: Supplementary file 2 — Data S1: lary70467‐sup‐0001‐Supinfo.pdf. [file LARY-136-3225-s001.pdf]

PATIENT CONSENT FORM FOR USE OF PHOTOGRAPHS

I, FONG YAM Bettina, being a person of sound mind, hereby give my permission to include Doctor GARGULA Stéphane identifiable photograph(s) of me in [title of work] (the "Work") to be published by Wiley-Blackwell ("Wiley"). This permission extends to all editions and versions (including all language versions) of the Work to be published by Wiley or its licensees throughout the world, in all media of expression now known or later developed.

I declare, in consequence of granting this permission, that I have no claim on ground of breach of confidence or on any ground in any legal system against Doctor GARGULA Stéphane in respect of the publication of the photograph(s).

In cases where the patient has died or is incapable of giving consent, consent may be given by the next of kin. If the patient is under the age of 16, consent should be given by a parent or guardian.

NAME OF PATIENT: FONG YAM Bettina
SIGNATURE OF PATIENT
………………………………………………
ADDRESS: C/O PRIORI BP 273 Antananarivo MADAGASCAR

08/01/2026

[NAME OF DOCTOR]
SIGNATURE OF DOCTOR ………………………………………
[ADDRESS]

[DATE]
